# Association of Thyroid Hormone Treatment Intensity With Cardiovascular Mortality Among US Veterans

**DOI:** 10.1001/jamanetworkopen.2022.11863

**Published:** 2022-05-12

**Authors:** Josh M. Evron, Scott L. Hummel, David Reyes-Gastelum, Megan R. Haymart, Mousumi Banerjee, Maria Papaleontiou

**Affiliations:** 1 Division of Endocrinology and Metabolism, Department of Internal Medicine, University of North Carolina, Chapel Hill; 2 Division of Cardiovascular Medicine, Department of Internal Medicine, University of Michigan, Ann Arbor; 3 Division of Metabolism, Endocrinology and Diabetes, Department of Internal Medicine, University of Michigan, Ann Arbor; 4 School of Public Health, Department of Biostatistics, University of Michigan, Ann Arbor; 5 Institute of Gerontology, University of Michigan, Ann Arbor

## Abstract

**Importance:**

Cardiovascular disease is the leading cause of death in the United States. Synthetic thyroid hormones are among the 3 most commonly prescribed medications, yet studies evaluating the association between the intensity of thyroid hormone treatment and cardiovascular mortality are scarce.

**Objective:**

To evaluate the association between thyroid hormone treatment intensity and cardiovascular mortality.

**Design, Setting, and Participants:**

This retrospective cohort study used data on 705 307 adults who received thyroid hormone treatment from the Veterans Health Administration Corporate Data Warehouse between January 1, 2004, and December 31, 2017, with a median follow-up of 4 years (IQR, 2-9 years). Two cohorts were studied: 701 929 adults aged 18 years or older who initiated thyroid hormone treatment with at least 2 thyrotropin measurements between treatment initiation and either death or the end of the study period, and, separately, 373 981 patients with at least 2 free thyroxine (FT_4_) measurements. Data were merged with the National Death Index for mortality ascertainment and cause of death, and analysis was conducted from March 25 to September 2, 2020.

**Exposures:**

Time-varying serum thyrotropin and FT_4_ levels (euthyroidism: thyrotropin level, 0.5-5.5 mIU/L; FT_4_ level, 0.7-1.9 ng/dL; exogenous hyperthyroidism: thyrotropin level, <0.5 mIU/L; FT_4_ level, >1.9 ng/dL; exogenous hypothyroidism: thyrotropin level, >5.5 mIU/L; FT_4_ level, <0.7 ng/dL).

**Main Outcomes and Measures:**

Cardiovascular mortality (ie, death from cardiovascular causes, including myocardial infarction, heart failure, or stroke). Survival analyses were performed using Cox proportional hazards regression models using serum thyrotropin and FT_4_ levels as time-varying covariates.

**Results:**

Of the 705 307 patients in the study, 625 444 (88.7%) were men, and the median age was 67 years (IQR, 57-78 years; range, 18-110 years). Overall, 75 963 patients (10.8%) died of cardiovascular causes. After adjusting for age, sex, traditional cardiovascular risk factors (eg, hypertension, smoking, and previous cardiovascular disease or arrhythmia), patients with exogenous hyperthyroidism (eg, thyrotropin levels, <0.1 mIU/L: adjusted hazard ratio [AHR], 1.39; 95% CI, 1.32-1.47; FT_4_ levels, >1.9 ng/dL: AHR, 1.29; 95% CI, 1.20-1.40) and patients with exogenous hypothyroidism (eg, thyrotropin levels, >20 mIU/L: AHR, 2.67; 95% CI, 2.55-2.80; FT_4_ levels, <0.7 ng/dL: AHR, 1.56; 95% CI, 1.50-1.63) had increased risk of cardiovascular mortality compared with individuals with euthyroidism.

**Conclusions and Relevance:**

This study suggests that both exogenous hyperthyroidism and exogenous hypothyroidism were associated with increased risk of cardiovascular mortality. These findings emphasize the importance of maintaining euthyroidism to decrease cardiovascular risk and death among patients receiving thyroid hormone treatment.

## Key Points

**Question:**

Is there an association between the intensity of thyroid hormone treatment and cardiovascular mortality?

**Findings:**

In this population-based cohort study of 705 307 adults who received thyroid hormone treatment, 10.8% died of cardiovascular causes. Both exogenous hyperthyroidism and exogenous hypothyroidism were associated with increased risk of cardiovascular mortality after adjusting for a comprehensive set of demographic and traditional cardiovascular risk factors.

**Meaning:**

These findings suggest that the intensity of thyroid hormone treatment may be a modifiable risk factor for cardiovascular mortality.

## Teaser

This cohort study uses data from the Veterans Health Administration Corporate Data Warehouse database to evaluate the association between the intensity of thyroid hormone treatment and cardiovascular mortality.

## Introduction

Despite widespread efforts at prevention and advances in diagnosis and treatment, cardiovascular disease remains the leading cause of death in the United States and affects nearly 50% of individuals in the US aged 20 years or older.^1,2^ In addition, it is estimated that the annual financial burden of heart disease in the United States is more than $200 billion.^1^ Although many cardiovascular risk factors are known (eg, hypertension, diabetes, and smoking), the persistent public health impact of cardiovascular disease mandates a more complete understanding of novel risk factors.^1,3^ A recent study has shown that the intensity of thyroid hormone treatment is a modifiable risk factor for incident atrial fibrillation and stroke^4^; however, its association with cardiovascular mortality remains unclear.

Thyroid hormone treatment is widespread, with levothyroxine prescriptions consistently among the top 3 of all prescription medications in the United States in the past decade.^5,6,7^ However, up to 50% of patients who receive thyroid hormone treatment may exhibit exogenous hyperthyroidism or hypothyroidism (ie, have thyrotropin levels below or above the reference range, respectively).^8,9^ The associations of long-term exogenous hyperthyroidism and hypothyroidism with clinical outcomes, including cardiovascular risk and all-cause mortality, have recently been investigated.^4,10,11,12,13^ Previous studies have shown that serum thyrotropin concentrations outside the euthyroid range correlated with increased cardiovascular risk and all-cause mortality among patients who received thyroid hormone treatment for hypothyroidism^11,13,14^; however, studies focusing specifically on the association between the intensity of thyroid hormone treatment and cardiovascular mortality are lacking.

The objective of this study was to evaluate the association between the intensity of thyroid hormone treatment and cardiovascular mortality using a nationwide, population-based cohort of adults receiving thyroid hormone treatment. We hypothesized that both exogenous hyperthyroidism and hypothyroidism would be associated with increased cardiovascular mortality, even when adjusting for age, sex, and traditional cardiovascular risk factors, such as hypertension and smoking.

## Methods

### Data Source and Study Population

We conducted a population-based, retrospective cohort study between January 1, 2004, and December 31, 2017, using data from the Veterans Health Administration.^15^ This large, integrated health care system provides care to more than 9 million US veterans annually.^16^ Deidentified patient-level data were obtained using the Veterans Health Administration Corporate Data Warehouse database, which is a national centralized data repository for the Veterans Health Administration. This database provides clinical and administrative information, including diagnoses, laboratory data, pharmacy prescription fills, health factors, and demographic information.^17,18,19^ These data were then linked to the National Death Index for mortality ascertainment and to identify cause of death.^20^ The study followed the Strengthening the Reporting of Observational Studies in Epidemiology (STROBE) reporting guideline.^21^ This study was exempt from the University of Michigan institutional review board and approved by the Ann Arbor Veteran Affairs institutional review board, which included a waiver of informed consent as data were deidentified.

The study population included 705 307 patients aged 18 years or older who initiated thyroid hormone treatment during the study period. Of these patients, 701 929 had at least 2 outpatient measurements of serum thyrotropin between initiation of thyroid hormone treatment and either death or the end of the study. We also studied patients aged 18 years or older receiving thyroid hormone treatment who had at least 2 outpatient free thyroxine (FT_4_) measurements between initiation of thyroid hormone treatment and death or study conclusion (n = 373 981). Patients with a history of thyroid cancer (n = 15 090) were excluded because lower thyrotropin levels are often targeted to reduce the risk of disease recurrence. In addition, patients prescribed lithium (n = 23 715) or amiodarone (n = 70 358) were excluded because these medications have a known association with abnormal thyroid function test results. Patients who did not have a documented date of birth (n = 17) were also excluded prior to attaining the final analytic sample.

### Measures

#### Study Outcome

The study outcome was cardiovascular mortality as determined by cause of death from cardiovascular diseases based on *International Statistical Classification of Diseases and Related Health Problems, Tenth Revision* (*ICD-10*) codes (including codes I00-I99). The incident event (ie, death from cardiovascular causes) occurred after initiation of thyroid hormone treatment and through December 31, 2017, with a median follow-up of 4 years (IQR, 2-9 years).

#### Exposure Variables

The exposure variables were time-varying serum thyrotropin and FT_4_ levels. Serum thyrotropin and FT_4_ levels were compiled using the patients’ laboratory records and assembled into 2 separate longitudinal data sets. Prior to analyses, thyrotropin levels were log transformed owing to nonnormal distribution, as has been done in prior studies cited in the literature.^4,10,22^ Although there is some variation by laboratory, the reference ranges for thyrotropin and FT_4_ levels at the Ann Arbor VA laboratory were used for analyses (thyrotropin level, 0.5-5.5 mIU/L; FT_4_ level, 0.7-1.9 ng/mL [to convert to picomoles per liter, multiply by 12.87]). Of note, 99.1% of patients (370 603 of 373 981) who had at least 2 FT_4_ measurements also had at least 2 thyrotropin measurements.

#### Covariates

Fixed covariates included patient sex, age, race, ethnicity, and smoking status. Data on sex were obtained from the Veterans Health Administration Corporate Data Warehouse database at study entry and recorded as male or female. Age was analyzed both as a continuous variable and as a categorical variable using clinically meaningful categories (18-49, 50-64, 65-74, 75-84, and ≥85 years). Race was self-reported as Alaska Native or American Indian, Asian, Black, Native Hawaiian or Pacific Islander, White, multiracial, or unknown. Because the number of patients who identified as Alaska Native or American Indian, Asian, Native Hawaiian or Pacific Islander, or multiracial was small, these groups were collapsed and classified as “other” for analyses. Ethnicity was self-described as Hispanic, non-Hispanic, or unknown. Smoking status was determined at the time of initiation of thyroid hormone treatment and was recorded as never smoker, current or former smoker, or unknown. Time-varying covariates included hypertension, hyperlipidemia, diabetes, prior history of cardiovascular disease (coronary artery disease, ischemic heart disease, heart failure, or stroke), and prior history of cardiac arrhythmia and were determined by using *International Classification of Diseases, Ninth Revision*; *International Statistical Classification of Diseases and Related Health Problems, Tenth Revision*; and *Current Procedure Terminology, Fourth Edition* codes.

### Statistical Analysis

Data were analyzed from March 25 to September 2, 2020. Descriptive information was tabulated for both the thyrotropin and FT_4_ cohorts. Univariate associations between individual factors and the outcome were examined. We then performed separate survival analyses using Cox proportional hazards regression models to determine correlates for the study outcome (ie, cardiovascular mortality). In these models, the exposure variables (thyrotropin and FT_4_ levels) were treated as time-varying covariates, which allowed us to better account for variability in these measures during the study period. Serum thyrotropin and FT_4_ levels were treated as categorical variables. Exogenous hyperthyroidism was defined by thyrotropin levels lower than 0.5 mIU/L (categorized as <0.1 mIU/L and 0.1-0.5 mIU/L) or by FT_4_ levels higher than 1.9 ng/dL; euthyroidism was defined by thyrotropin levels from 0.5 to 5.5 mIU/L and FT_4_ levels from 0.7 to 1.9 ng/dL; and exogenous hypothyroidism was defined by thyrotropin levels higher than 5.5 mIU/L (categorized as >5.5 to <7.5 mIU/L, 7.5 to <10 mIU/L, 10-20 mIU/L, and >20 mIU/L) or by FT_4_ levels lower than 0.7 ng/dL. When multiple measurements were present in an annual time period (defined as a calendar year), which occurred for 5.9% of the thyrotropin cohort (41 414 of 701 929) and 2.7% of the FT_4_ cohort (10 097 of 373 981), the geometric mean was calculated for the thyrotropin cohort, and the arithmetic mean was calculated for the FT_4_ cohort; these values were used for analyses. For both models, additional fixed covariates included patient sex, age, race, ethnicity, and smoking status, and additional time-varying covariates included hypertension, hyperlipidemia, diabetes, prior history of cardiovascular disease, and prior history of cardiac arrhythmia. No observations were excluded from the statistical analyses owing to missing information.

All statistical analyses were conducted using SAS, version 7.15 HF8 (SAS Institute Inc). A 95% CI was used to determine statistical significance, and *P* < .05 from 2-sided tests was considered statistically significant for all analyses. Model adequacy was assessed using generalized residuals-based diagnostics in SAS PROC PHREG.

## Results

Table 1 provides key demographic, comorbidity, and mortality data for the 705 307 patients receiving thyroid hormone treatment. Most patients were male (625 444 [88.7%]), White (559 173 [79.3%]), non-Hispanic (609 537 [86.4%]), had a history of hypertension (582 061 [82.5%]) or hyperlipidemia (582 389 [82.6%]), and were current or former smokers (467 606 [66.3%]). The median age was 67 years (IQR, 57-78 years; range, 18-110 years). During the study period, 224 943 patients (31.9%) died of any cause, and 75 963 patients (10.8%) died of cardiovascular disease.

**Table 1.** Characteristics of Patients Receiving Thyroid Hormone Therapy

Characteristic	Patients, No. (%)		
	All patients (N = 705 307)	Patients with at least 2 thyrotropin measurements (n = 701 929)	Patients with at least 2 free thyroxine measurements (n = 373 981)^a^
Sex			
Male	625 444 (88.7)	622 396 (88.7)	325 217 (87.0)
Female	79 863 (11.3)	79 533 (11.3)	48 764 (13.0)
Age, y			
18-49	89 765 (12.7)	89 462 (12.7)	57 046 (15.2)
50-64	224 876 (31.9)	223 986 (31.9)	133 202 (35.6)
65-74	169 177 (24.0)	168 408 (24.0)	85 917 (23.0)
75-84	165 944 (23.5)	164 939 (23.5)	74 762 (20.0)
≥85	55 545 (7.9)	55 134 (7.9)	23 054 (6.2)
Race			
Black	49 535 (7.0)	49 113 (7.0)	31 219 (8.3)
White	559 173 (79.3)	556 879 (79.3)	299 043 (80.0)
Other^b^	15 903 (2.3)	15 833 (2.3)	8594 (2.3)
Unknown	80 696 (11.4)	80 104 (11.4)	35 125 (9.4)
Ethnicity			
Hispanic	37 740 (5.4)	37 636 (5.4)	20 685 (5.5)
Non-Hispanic	609 537 (86.4)	606 759 (86.4)	328 766 (87.9)
Unknown	58 030 (8.2)	57 534 (8.2)	24 530 (6.6)
Smoking			
Never	92 057 (13.1)	91 757 (13.1)	56 815 (15.2)
Current or former	467 606 (66.3)	465 392 (66.3)	248 501 (66.4)
Unknown	145 644 (20.7)	144 780 (20.6)	68 665 (18.4)
Hypertension	582 061 (82.5)	579 453 (82.6)	308 899 (82.6)
Hyperlipidemia	582 389 (82.6)	580 049 (82.6)	313 044 (83.7)
Diabetes	302 641 (42.9)	301 407 (42.9)	163 373 (43.7)
Prior history of cardiovascular disease	114 534 (16.2)	113 993 (16.2)	65 453 (17.5)
Prior history of cardiac arrhythmia	184 822 (26.2)	184 167 (26.2)	102 573 (27.4)
Study outcome			
Death from cardiovascular causes	75 963 (10.8)	75 319 (10.7)	33 142 (8.9)

^a^ There were 99.1% of patients who had at least 2 free thyroxine measurements and also at least 2 thyrotropin measurements.

^b^ Composed of the following races: Alaska Native or American Indian, Asian, Native Hawaiian or Pacific Islander, and multiracial.

The frequency distributions of the number of thyrotropin measurements and the number of FT_4_ measurements overall and in association with the number of years, as well as the mean number of thyrotropin measurements and FT_4_ measurements per patient per year, are shown in eTable 1 and eFigures 1 and 2 in the Supplement. In univariate analyses, patient sex, age, race, ethnicity, smoking status, hypertension, hyperlipidemia, diabetes, prior history of cardiovascular disease, prior history of cardiac arrhythmia, and serum thyrotropin and FT_4_ levels were significantly associated with cardiovascular mortality.

Table 2 shows results from survival analyses using Cox proportional hazards regression models demonstrating patient characteristics associated with cardiovascular mortality for the thyrotropin and FT_4_ cohorts. When adjusting for age, sex, and other relevant demographic and traditional cardiovascular risk factors (such as hypertension and smoking), patients with exogenous hyperthyroidism (eg, thyrotropin levels <0.1 mIU/L: adjusted hazard ratio [AHR], 1.39; 95% CI, 1.32-1.47; FT_4_ levels >1.9 ng/dL: AHR, 1.29; 95% CI, 1.20-1.40) and patients with exogenous hypothyroidism (eg, thyrotropin levels >20 mIU/L: AHR, 2.67; 95% CI, 2.55-2.80; FT_4_ levels <0.7 ng/dL: AHR, 1.56; 95% CI, 1.50-1.63) had an increased risk of cardiovascular mortality compared with individuals with euthyroidism. Furthermore, the risk of cardiovascular mortality increased progressively with lower and higher thyrotropin levels compared with euthyroidism. Similar findings were obtained when age was treated as a continuous variable (eTable 2 in the Supplement).

**Table 2.** Characteristics of Patients Receiving Thyroid Hormone Therapy Associated With Cardiovascular Mortality

Patient characteristic	Adjusted hazard ratio (95% CI)
**Thyrotropin cohort**	
Thyrotropin level (annual geometric mean), mIU/L	
<0.1	1.39 (1.32-1.47)
0.1 to <0.5	1.13 (1.09-1.17)
0.5 to 5.5	1 [Reference]
>5.5 to <7.5	1.42 (1.38-1.46)
7.5 to <10	1.76 (1.70-1.82)
10 to 20	2.13 (2.05-2.21)
>20	2.67 (2.55-2.80)
Sex	
Male	1 [Reference]
Female	0.68 (0.66-0.71)
Age, y	
18-49	1 [Reference]
50-64	2.87 (2.68-3.07)
65-74	5.97 (5.59-6.38)
75-84	14.55 (13.63-15.53)
≥85	27.40 (25.62-29.29)
Race	
Black	0.96 (0.93-1.00)
White	1 [Reference]
Other^a^	0.90 (0.85-0.95)
Unknown	1.34 (1.30-1.37)
Ethnicity	
Hispanic	0.65 (0.62-0.67)
Non-Hispanic	1 [Reference]
Unknown	1.74 (1.69-1.78)
Smoking	
Never	1 [Reference]
Current or former	1.22 (1.19-1.25)
Unknown	1.52 (1.48-1.56)
Hypertension	1.60 (1.56-1.65)
Hyperlipidemia	0.92 (0.90-0.93)
Diabetes	1.41 (1.38-1.43)
Prior history of cardiovascular disease	1.40 (1.38-1.42)
Prior history of cardiac arrhythmia	1.97 (1.94-2.00)
**Free thyroxine cohort**	
Free thyroxine level (annual arithmetic mean), ng/dL^b^	
<0.7	1.56 (1.50-1.63)
0.7-1.9	1 [Reference]
>1.9	1.29 (1.20-1.40)
Sex	
Male	1 [Reference]
Female	0.64 (0.60-0.67)
Age, y	
18-49	1 [Reference]
50-64	2.50 (2.29-2.72)
65-74	4.98 (4.58-5.43)
75-84	11.81 (10.86-12.84)
≥85	20.44 (18.74-22.28)
Race	
Black	0.93 (0.89-0.98)
White	1 [Reference]
Other^a^	0.98 (0.90-1.06)
Unknown	1.36 (1.31-1.41)
Ethnicity	
Hispanic	0.65 (0.61-0.69)
Non-Hispanic	1 [Reference]
Unknown	1.64 (1.57-1.71)
Smoking	
Never	1 [Reference]
Current or former	1.22 (1.18-1.26)
Unknown	1.44 (1.38-1.49)
Hypertension	1.75 (1.68-1.82)
Hyperlipidemia	0.95 (0.92-0.98)
Diabetes	1.41 (1.37-1.44)
Prior history of cardiovascular disease	1.47 (1.43-1.50)
Prior history of cardiac arrhythmia	2.07 (2.02-2.12)

^a^ Composed of the following races: Alaska Native or American Indian, Asian, Native Hawaiian or Pacific Islander, and multiracial.

^b^ To convert free thyroxine to picomoles per liter, multiply by 12.87.

The forest plot in the Figure illustrates the association between serum thyrotropin and FT_4_ levels with cardiovascular mortality after adjustment for relevant demographic and cardiovascular risk factors. Cardiovascular mortality was higher among patients with exogenous hyperthyroidism (thyrotropin levels <0.1 mIU/L: AHR, 1.39; 95% CI, 1.32-1.47; thyrotropin levels of 0.1 to <0.5 mIU/L: AHR, 1.13; 95% CI, 1.09-1.17; FT_4_ levels >1.9 ng/dL: AHR, 1.29; 95% CI, 1.20-1.40) and those with exogenous hypothyroidism (thyrotropin levels from >5.5 to <7.5 mIU/L: AHR, 1.42; 95% CI, 1.38-1.46; thyrotropin levels from 7.5 to <10 mIU/L: AHR, 1.76; 95% CI, 1.70-1.82; thyrotropin levels of 10-20 mIU/L: AHR, 2.13; 95% CI, 2.05-2.21; thyrotropin levels >20 mIU/L: AHR, 2.67; 95% CI, 2.55-2.80; FT_4_ levels <0.7 ng/dL: AHR, 1.56; 95% CI, 1.50-1.63), with risk increasing with higher serum thyrotropin levels compared with individuals with euthyroidism.

**Figure.**
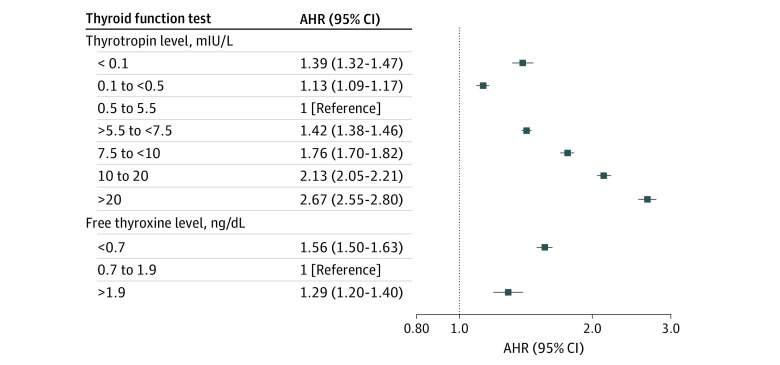
Association of Thyrotropin and Free Thyroxine Levels With Cardiovascular Mortality This forest plot illustrates the association of serum thyrotropin and free thyroxine levels with cardiovascular mortality after adjustment for relevant demographic and cardiovascular risk factors. To convert free thyroxine to picomoles per liter, multiply by 12.87. AHR indicates adjusted hazard ratio.

## Discussion

In this population-based study of a large cohort of adults receiving thyroid hormone treatment, we found that the intensity of thyroid hormone treatment was associated with cardiovascular mortality. Both exogenous hyperthyroidism and hypothyroidism were associated with increased risk of cardiovascular mortality, even when adjusting for relevant demographic and traditional cardiovascular risk factors as well as previous history of cardiovascular disease and/or arrhythmia. In addition, our findings suggest that the risk of cardiovascular mortality is directly associated with the degree of thyrotropin abnormality outside the euthyroid range, such that thyrotropin levels lower than 0.1 mIU/L and higher than 20 mIU/L were associated with the highest increased risk. We observed that the AHRs increased more rapidly for older age categories in the thyrotropin cohort compared with the FT_4_ cohort. This finding may suggest that the thyrotropin level is more strongly associated with cardiovascular risk than is the FT_4_ level in older adults. From a clinical perspective, older adults, and particularly the oldest old (aged ≥85 years), appear to be the most vulnerable, with increased risk of cardiovascular mortality with both exogenous hyperthyroidism and hypothyroidism.

A few prior studies have evaluated the association between serum thyrotropin levels and cardiovascular outcomes among patients receiving thyroid hormone treatment.^4,10,11,13^ Flynn et al^10^ used a time-weighted mean thyrotropin level to group patients and demonstrated increased risk of a composite outcome of cardiovascular admission or death with suppressed or elevated serum thyrotropin levels in a cohort of 17 684 patients treated with levothyroxine. In addition, Lillevang-Johansen et al^11^ performed a case-control study of 20 487 patients with incident cardiovascular disease nested within a larger cohort of individuals with hypothyroidism and showed that, compared with matched controls, patients with treated hypothyroidism (n = 636) had increased odds of incident cardiovascular disease and all-cause mortality for each 6-month period of overtreatment or undertreatment. Finally, Thayakaran et al^13^ evaluated the association between thyrotropin concentration and incident cardiovascular outcomes, but not mortality, among patients with hypothyroidism (N = 160 439), adjusting for levothyroxine treatment. This study found that compared with a thyrotropin level of 2 to 2.5 mIU/L, a thyrotropin level higher than 10 mIU/L was associated with an increased risk of ischemic heart disease and heart failure. None of these studies evaluated the association of the intensity of thyroid hormone treatment with cardiovascular mortality, possibly because of inadequate power. Our study is novel because, unlike these prior studies, it specifically evaluated the association between cardiovascular mortality and serum thyrotropin or FT_4_ level, while adjusting for a comprehensive list of possible confounders, exclusively in a large cohort of patients receiving thyroid hormone treatment.

Our study findings have the potential to affect how we think about the risks and benefits associated with thyroid hormone treatment, particularly for vulnerable populations, such as older adults or those with underlying cardiovascular disease. Because synthetic thyroid hormones have consistently been one of the top 3 most frequently prescribed medications in the United States in the past decade,^5,6,7^ and because cardiovascular disease remains the leading cause of death, an association between the intensity of thyroid hormone treatment and cardiovascular death has far-reaching implications for both patients and physicians. Although the variability in thyrotropin and FT_4_ levels and thyroid hormone dose adjustments are an inevitable reality for most patients, our study emphasizes the importance of regular monitoring of thyroid function test results and correction of both overtreatment and undertreatment with exogenous thyroid hormones to reduce patient harm, particularly for older adults who are at higher risk for adverse effects.^4,8,9,23^

### Strengths and Limitations

Our study has several strengths. First, it is a large, population-based study using data from the largest integrated health care system in the United States. Second, we used serum thyrotropin and FT_4_ levels as time-varying covariates, which allowed us to incorporate all qualifying thyrotropin and FT_4_ levels measured during the study period. Because prior studies have shown wide variability in thyroid function test results over time among patients receiving thyroid hormone treatment,^8,9,24,25^ this method facilitates a more comprehensive evaluation of the association between thyroid function test results and cardiovascular mortality than would be possible using a single value at study entry in a cross-sectional design. Third, while the use of an internal comparator cannot completely eliminate risk of confounding, it avoids the inherent limitations of comparing an exposed group with the background population. Fourth, because the Veterans Health Administration Corporate Data Warehouse includes comprehensive information on patient demographic characteristics, comorbid conditions, and smoking status, we were able to account for most traditional cardiovascular risk factors.

Population-based studies using databases have some inherent limitations that merit consideration. Although we were able to account for most known cardiovascular risk factors, we were not able to control for other potential confounders, such as alcohol status and body mass index or rates of obesity, because these could not be accurately captured in the Veterans Health Administration data. Adjusting for a comprehensive list of cardiovascular risk factors, including hypertension, hyperlipidemia, diabetes, and prior history of cardiovascular disease, which represent some of the downstream effects of obesity, partially mitigates this limitation. In addition, we were unable to determine the degree to which risk factors for cardiovascular mortality, such as diabetes and hypertension, were appropriately treated. We also acknowledge that we were not able to account for all medications and supplements that could interfere with thyroid hormone metabolism and action and/or thyroid function test results. Furthermore, because cause of death in the National Death Index relies on death certificates, risk of misclassification is possible.^26^ Because the Veterans Health Administration population is predominantly male, women are generally underrepresented in studies using this database. However, because the risk of cardiovascular disease is higher for men than for women^27^ and because more than 70 000 women were included in this cohort, the results of this study are highly clinically relevant. Additionally, this was an observational study, and a causal relationship between the intensity of thyroid hormone treatment and cardiovascular mortality could not be definitively established.

## Conclusions

In this population-based cohort of patients receiving thyroid hormone treatment, we found that both exogenous hyperthyroidism and hypothyroidism were associated with an increased risk of cardiovascular mortality after adjusting for a comprehensive set of demographic and traditional cardiovascular risk factors. Cardiovascular disease remains the leading cause of death in the United States, and its economic impact is enormous. Identifying and addressing modifiable risk factors continues to be critically important to reducing the rates of cardiovascular disease and mortality. The emergence of the intensity of thyroid hormone treatment as a potential associated risk factor provides a highly relevant and easily modifiable clinical parameter for patients who receive thyroid hormone treatment.

## Notes

Supplement. **eTable 1.** Frequency Distributions of the Number of Thyrotropin and Free Thyroxine Measurements **eFigure 1.** Mean Number of Thyrotropin Measurements per Patient, by Year **eFigure 2.** Mean Number of Free Thyroxine Measurements per Patient, by Year **eTable 2.** Characteristics of Patients on Thyroid Hormone Therapy Associated With Cardiovascular Mortality (Age as a Continuous Variable)
