# Supplementary material for: Association of Thyroid Hormone Treatment Intensity With Cardiovascular Mortality Among US Veterans
Source: JAMA Netw Open. 2022 May 12;5(5):e2211863. doi: 10.1001/jamanetworkopen.2022.11863 (PMC9099430; doi:10.1001/jamanetworkopen.2022.11863)
Supplement: Supplement. — eTable 1. Frequency Distributions of the Number of Thyrotropin and Free Thyroxine Measurements eFigure 1. Mean Number of Thyrotropin Measurements per Patient, by Year eFigure 2. Mean Number of Free Thyroxine Measurements per Patient, by Year eTable 2. Characteristics of Patients on Thyroid Hormone Therapy Associated With Cardiovascular Mortality (Age as a Continuous Variable) [file jamanetwopen-e2211863-s001.pdf]

Supplementary Online Content

Evron JM, Hummel SL, Reyes-Gastelum D, Haymart MR, Banerjee M, Papaleontiou M. Association of thyroid hormone treatment intensity with cardiovascular mortality among US veterans. *JAMA Netw Open.* 2022;5(5):e2211863. doi:10.1001/jamanetworkopen.2022.11863

eTable 1. Frequency Distributions of the Number of Thyrotropin and Free Thyroxine Measurements
eFigure 1. Mean Number of Thyrotropin Measurements per Patient, by Year
eFigure 2. Mean Number of Free Thyroxine Measurements per Patient, by Year
eTable 2. Characteristics of Patients on Thyroid Hormone Therapy Associated With Cardiovascular Mortality (Age as a Continuous Variable)

This supplementary material has been provided by the authors to give readers additional information about their work.

© 2022 Evron JM et al. *JAMA Network Open.*

eTable 1. Frequency Distributions of the Number of Thyrotropin and Free Thyroxine Measurements

	**Thyrotropin**	**Free Thyroxine**
**Total number of measurements**	1,760,141	585,929
**Number of measurements by year**		
2004	50,714	14,886
2005	80,961	22,797
2006	89,750	25,594
2007	92,083	26,993
2008	95,120	29,174
2009	99,227	30,714
2010	103,940	31,905
2011	107,376	34,471
2012	112,290	37,457
2013	116,079	39,544
2014	123,807	43,457
2015	134,730	51,976
2016	165,679	65,428
2017	388,385	131,533

© 2022 Evron JM et al. *JAMA Network Open.*

eFigure 1. Mean Number of Thyrotropin Measurements per Patient, by Year

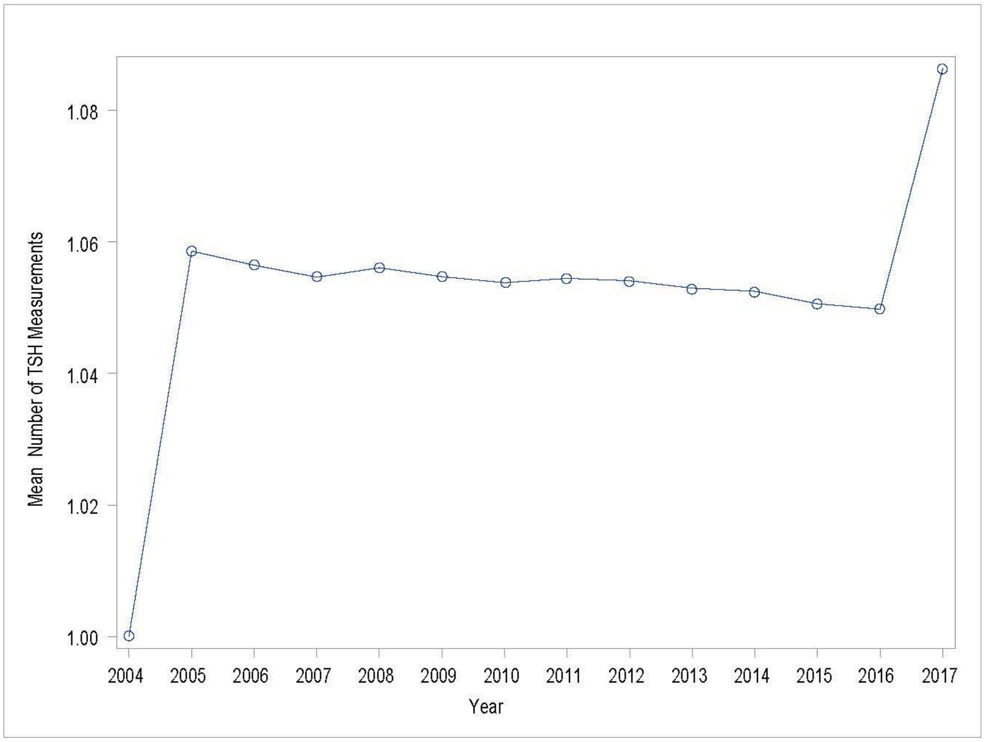

© 2022 Evron JM et al. *JAMA Network Open.*

eFigure 2. Mean Number of Free Thyroxine Measurements per Patient, by Year

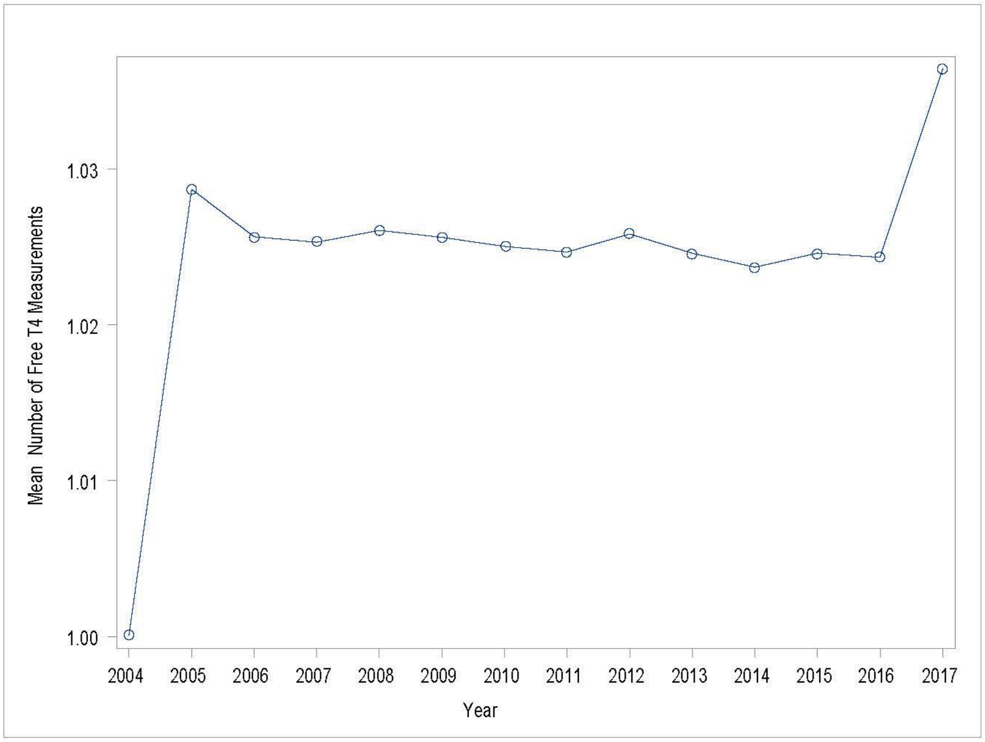

© 2022 Evron JM et al. *JAMA Network Open.*

eTable 2. Characteristics of Patients on Thyroid Hormone Therapy Associated With Cardiovascular Mortality (Age as a Continuous Variable)

Thyrotropin Cohort		Free Thyroxine Cohort	
**Patient Characteristics**	**Adjusted Hazard Ratio (95%CI)**	**Patient Characteristics**	**Adjusted Hazard Ratio (95%CI)**
Thyrotropin (annual geometric mean)		Free Thyroxine (annual arithmetic mean)	
< 0.1	1.42 (1.34–1.50)	<0.7	1.59 (1.53–1.66)
0.1 – <0.5	1.14 (1.10–1.18)	0.7–1.9	1 [Reference]
0.5 – 5.5	1 [Reference]	>1.9	1.29 (1.19–1.40)
>5.5 – <7.5	1.41 (1.38–1.45)		
7.5 – <10	1.76 (1.70–1.83)		
10 – 20	2.16 (2.08–2.24)		
> 20	2.75 (2.62–2.88)		
Sex		Sex	
Male	1 [Reference]	Male	1 [Reference]
Female	0.72 (0.69–0.75)	Female	0.68 (0.64–0.72)
Age (years)	1.08 (1.08-1.08)	Age (years)	1.08 (1.08-1.08)
Race		Race	
White	1 [Reference]	White	1 [Reference]
Black	0.99 (0.96–1.03)	Black	0.96 (0.92–1.01)
Other[a]	0.91 (0.86–0.97)	Other	0.99 (0.91–1.07)
Unknown	1.33 (1.29–1.36)	Unknown	1.35 (1.30–1.41)
Ethnicity		Ethnicity	
Not Hispanic	1 [Reference]	Not Hispanic	1 [Reference]
Hispanic	0.65 (0.62–0.68)	Hispanic	0.65 (0.62–0.69)
Unknown	1.74 (1.69–1.78)	Unknown	1.64 (1.57–1.71)
Smoking		Smoking	
Never	1 [Reference]	Never	1 [Reference]
Current/Former	1.22 (1.19–1.25)	Current/Former	1.22 (1.19–1.26)
Unknown	1.51 (1.47–1.54)	Unknown	1.42 (1.37–1.47)
Hypertension	1.55 (1.50–1.59)	Hypertension	1.67 (1.60–1.74)
Hyperlipidemia	0.93 (0.91–0.95)	Hyperlipidemia	0.96 (0.93–0.99)
Diabetes mellitus	1.42 (1.40–1.44)	Diabetes mellitus	1.42 (1.38–1.45)
Prior history of cardiovascular disease	1.39 (1.37–1.42)	Prior history of cardiovascular disease	1.46 (1.42–1.49)
Prior history of cardiac arrhythmia	1.92 (1.89–1.95)	Prior history of cardiac arrhythmia	2.02 (1.98–2.07)

[a] The category “Other” is composed of the following races: Asian, Native Hawaiian or Pacific Islander, Alaska Native or American Indian, and multiracial.
Units: mIU/L (thyrotropin), ng/dL (free thyroxine)

© 2022 Evron JM et al. *JAMA Network Open.*
